# Angiogenesis and lymphangiogenesis in primary cutaneous lymphomas: a narrative review

**DOI:** 10.1007/s10238-025-01853-2

**Published:** 2025-10-31

**Authors:** Mario Della Mura, Joana Sorino, Gerardo Cazzato, Giuseppe Ingravallo, Alessio Giubellino, Domenico Ribatti

**Affiliations:** 1 Section of Molecular Pathology, Department of Precision and Regenerative Medicine and Ionian Area (DiMePRe-J), University of Bari “Aldo Moro”, 70124 Bari, Italy; 2 Department of Laboratory Medicine & Pathology, University of Minnesota, Minneapolis, MN 55455 USA; 3 Department of Translational Biomedicine and Neuroscience, University of Bari Medical School, Bari, Italy

**Keywords:** Primary cutaneous lymphoma, T cell lymphoma, B cell lymphoma, Mycosis fungoides, Angiogenesis, Microvessel density, Lymphangiogenesis

## Abstract

Angiogenesis and lymphangiogenesis are hallmarks of cancer and widely investigated both in solid and hematologic malignancies, correlating with tumor aggressiveness and progression. To date, the meaning of angiogenesis in primary cutaneous lymphomas has not been fully clarified, with most data coming from T cell lymphomas, in particular mycosis fungoides. Herein, we provide a comprehensive review about their significance in both primary cutaneous T cell and B cell lymphomas, with the aim to deepen the underlying molecular mechanism and role in tumor development and progression.

## Introduction

Primary cutaneous lymphomas (CLs) are defined as non-Hodgkin lymphomas presenting in the skin with no evidence of extracutaneous disease at the time of diagnosis [1]. In most cases, they remain limited to the skin over long periods, with extracutaneous spreading usually occurring only in advanced stages, differing from systemic lymphomas with similar histological, phenotypic and genetic features that potentially involve the skin secondarily [2]. Certain subtypes show considerable overlap in the clinical or pathological features, and they exhibit broad range of clinical behavior, including lesions that spontaneously regress, lymphoid proliferations that persist but pursue an indolent course and lesions that rapidly progress into aggressive malignancies [3].

Primary CL includes both T cell lymphomas (CTCLs) and cutaneous B cell lymphomas (CBCLs).

In both cutaneous and systemic lymphomas, a pro-angiogenic microenvironment is associated with more aggressive behavior and poorer prognosis [4–6]. In this context, we aim to further explore the role of neoangiogenesis and lymphangiogenesis in CTCLs and CBCLs by contextualizing these processes within their biological framework, elucidating the underlying molecular mechanisms, and identifying potential therapeutic targets.

## CTCL features

CTCL are a heterogeneous group of lymphoid malignancies derived from skin-homing T cells [7–10]. The most common subtypes are mycosis fungoides (MF) (~ 60%) and CD30^+^ lymphoproliferative disorders (~ 30%), followed by Sezary syndrome (SS) (2–3%) [2].

MF shows a classic clinical progression from erythematous patches to plaques and tumors [11,12]. Early lesions are characterized by epidermotropic CD4^+^ T cells with cerebriform nuclei; then, a prominent colonization of both upper layers of the epidermis, sometimes with clusters formation tightly associated with Langerhans cell scaffolding [13], as well as of the dermis in form of diffuse sheets is seen. Dermal fibrosis frequently accompanies the disease and is a hallmark of the chronicity of these lesions [14]. Rare variants include folliculotropic MF, pagetoid reticulosis, and granulomatous slack skin [2]. While MF is typically indolent, a subset progresses with extracutaneous spread or leukemic transformation into SS [11].

SS is a rare leukemic type of CTCL, defined by the triad of pruritic erythroderma, generalized lymphadenopathy, and clonally related neoplastic T cells with cerebriform nuclei (Sezary cells) in skin, lymph nodes and peripheral blood [1]. The histologic features of SS resemble those seen in MF. However, the superficial perivascular infiltrates may be sparse, epidermotropism may be minimal or absent, or the histological picture may be aspecific, sometimes resembling an inflammatory dermatosis [15]. Diagnosis relies on blood involvement criteria: absolute Sezary cell count > 1000/μL or specific immunophenotypic aberrations (e.g., CD4/CD8 ≥ 10, CD4 + /CD7 −  ≥ 40%) [1].

Although MF and SS differ in clinical behavior, recent single-cell studies suggest they may lie on a disease spectrum, driven by phenotypic shifts among T cell memory subsets[11,16,17]. Clonal evolution and intratumoral heterogeneity increase with disease stage: circulating malignant clones seed skin lesions and evolve genetically, leading to the emergence of new subclones, some of which may re-enter circulation [11,17].

The molecular landscape of MF and SS is complex and heterogeneous (non-disease-specific), with frequent copy number alterations and fewer recurrent somatic mutations [18]. Alterations in the NF-κB, DNA damage repair, and epigenetic pathways are common. NF-κB activation promotes T cell survival, while *TP53* and *KRAS* mutations are associated with poorer prognosis [18–25]. Furthermore, dysregulation of the JAK/STAT pathwaysupports tumor growth and pro-inflammatory cytokine production [26–30]. Epigenetic regulators such as *ARID1A*, *CTCF*, and *DNMT3A* are frequently deleted [31].

Primary cutaneous CD30^+^ T cell lymphoproliferative disorders include lymphomatoid papulosis (LyP) and primary cutaneous anaplastic large cell lymphoma (pcALCL). These entities share many histological and genetic features and often coexist. Furthermore, 10–20% of LyP patients also develop MF [32]. As a rule, neoplastic T cells express CD30 in all cases [2]. LyP presents with recurrent crops of papules and nodules following a characteristic waxing and waning course. It includes six subtypes [2,3,33], which differs histologically and immunophenotypically. NGS technology detected mutations that affect the IL-6-JAK1-STAT3 pathway in approximately 15–30% of cases; other frequent genetic alterations include mutations in *DNMT3A* and *TP53*, as well as in the PI3K and MAPK pathways [18].

pcALCL presents as a solitary nodule, without tendency to spontaneous resolution. Distinguishing PC-ALCL from skin involvement by systemic ALK ALCL remains a diagnostic challenge and requires the exclusion of extracutaneous involvement. *IRF4* locus gene rearrangement is seen in 20% to 75% of cases [34,35], while *ALK* rearrangement has traditionally been equated with systemic ALK-positive ALCL however, a small number ofcases of ALK-positive ALCL confined to the skin have been reported, predominantly in pediatric patients [36,37].

Some cutaneous lymphomas are classified as peripheral T cell lymphoma, not otherwise specified (PTCL-NOS), when they do not fulfill the diagnostic criteria of well-defined subtypes, even in the absence of extracutaneous involvement. These cases often exhibit a CD4 + phenotype, expression of follicular helper T cell markers, and are typically associated with an aggressive clinical course [3].

CTCLs are a heterogeneous group of lymphoid malignancies derived from skin-homing T cells, expressing receptors such as cutaneous lymphocyte antigen (CLA) and CC chemokine receptor 4 (CCR4) [7,38]. Accumulating evidence has asserted an increasingly role of malignant T cells in fostering the inflammatory milieu and fueling progressive immune dysregulation and tumor cell growth in CTCL patients, manifested through the secretion of Th2 cytokines and chemokines [11,13,39], almost in MF and SS.

In early disease, the tumor microenvironment (TME) is dominated by Th1/CD8^+^ cytotoxic responses [40,41]. With progression, malignant T cells adopt a Th2 phenotype, suggesting either TH2 cell skewing of TH1 cell-like malignant cells or preferential survival of TH 2 cell-like malignant cells with disease progression. Therefore, they secrete IL-4, IL-5, IL-10, IL-13, and IL-31 [7] that in their turn stimulate keratinocytes and fibroblasts to secrete Th2 chemokines, attracting more Th2 cells to the lesional skin. The Th2 bias of malignant T cells is intrinsic and fueled by cellular abnormalities or the secretion of autocrine growth factors, whereas the bias of non-clonal benign T cells is extrinsic and influenced by the production of Th2 cytokines by the malignant clones [11], thus creating a network that plays a critical role in maintaining and exacerbating the Th2-skewed TME, promoting the proliferation of malignant T cells and disease progression [13].

The driving force in mediating this shift toward a Th2-biased TME is represented by the gradual dysregulation of the JAK/STAT pathways [42–47]. Other TME componentscontribute to tumor progression [48–59]. IL-17Fis expressed constitutively in MF-associated malignant T cells and has been linked with the progressive disease state, as the consequence of induction of JAK/STAT3 pathway; in its turn, it controls levels of inflammatory and angiogenic factors [52].

Malignant T cells may also inhibit anti-tumor immunity by inducing apoptosis in reactive T cells, through Fas ligand (FasL) expression [60,61]]. Moreover, increased expression of immune checkpoint molecules on malignant T cells correlates positively with the advanced disease stage [29,62]. High levels of HMGB1 in skin lesions and serum are associated with increased Th2 immune response and induction of angiogenesis [63].

Skin barrier dysfunction plays also a key role in CTCL pathogenesis [64,65] Th2 cytokines reduce expression of structural proteins produced by keratinocytes, such as filaggrin (FLG), loricrin (LOR) and involucrin (IVL), along with antimicrobial peptides AMPs and keratin 10, leading to increased skin permeability and predisposing to microbial colonization [13,64,66]. Moreover, *Staphylococcus aureus* colonization and toxin production exacerbate inflammation and promote malignant T cell proliferation through STAT3/5 activation induced by enterotoxins. Finally, α-toxin contributes to immune evasion by inducing apoptosis of reactive T cells [13,67,68].

## CBCL features

CBCL are primary clonal proliferations derived from cutaneous B cellsThree main entities are recognized: primary cutaneous follicle center lymphoma (PCFCL), primary cutaneous marginal zone lymphoma (PCMZL), and primary cutaneous diffuse large B cell lymphoma, leg type (DLBCL-LT) [2,69].

PCFCL (~ 50% of CBCLs) presents as localized plaques or nodules, typically on the head, neck, or trunk. Histologically, it may show a follicular, follicular, and diffuse or diffuse growth pattern. Tumor cells express BCL6, other germinal center markers and variably CD10 (often lost in diffuse cases); Ki67 is typically > 30%. BCL2 is usually absent or faint [2,3,70]. Prognosis is excellent, and molecular features support derivation from germinal center B cells with somatic hypermutation [2,71].

PCMZL (30–40% of CBCLs) presents as solitary or clustered erythematous papules, plaques, or nodules. Histology shows dermal infiltrates of small B cells, plasma cells, and reactive follicles. Neoplastic cells are *BCL2*^+^, *CD5/CD10/BCL6/cyclin D1*^−^, with light chain restriction in plasma cells [2]. Two subtypes are recognized. The first one, accounting for the majority of PCMZL, consists of class-switched neoplasms, more frequently to IgG than IgA, constituted by a modest number of neoplastic cells with prominent plasmacytic differentiation. The second group consists of IgM-positive PCMZLs that more often involve the subcutis and contain a predominance of neoplastic monocytoid or centrocyte-like B cells in a less conspicuous background of reactive T cells Th1-shifted [3,72,73]. Overall, it exhibits remarkably indolent behavior, with extracutaneous dissemination exceedingly rare [3,74]. *FAS* mutations are found in the majority of patients, while *(t14;18)(q32;q21)/IGH::MALT1* rearrangement has been identified in up to one third of cases [2,75].

PCLBCL-LT (10–20%) affects mainly elderly women, presenting as fast-growing, ulcerated nodules most frequently on the legs, with tendency to systemic spread [2]. Biopsy shows diffuse sheets of large, atypical B cells, with a non-germinal center phenotype; EBV is negative. It originates from post-germinal center B cells and has a distinctive mutational profile, which overlaps significantly with that of primary CNS, primary testicular DLBCL, intravascular large B cell lymphoma and the MCD group of DLBCL, including a high frequency of MYD88 and CD79B mutations [3,76]. Highly recurrent hotspot mutations in the adaptor molecule of the Toll-like receptor MYD88 are found in 70–75% of cases.

## Mechanisms of tumor angiogenesis

It is well established that, to grow, sustain metabolic demands, survive, and metastasize, tumors must secure a constant supply of oxygen and nutrientsmade possible through the development of a dedicated vascular network, i.e., neoangiogenesis [4,77,78]. While historically foundational, this definition is now considered insufficient to reflect the full complexity of the process. Angiogenesis is recognized as a tightly orchestrated, multifactorial phenomenon involving a broad array of cellular contributors and molecular cross-talk that extends beyond the endothelial compartment [79]. Considering that tumors arise and evolve within a dynamic and interactive TME, malignant cells actively engage with stromal and immune components to reshape their niche. Within this evolving ecosystem, angiogenesis functions not merely as a supportive mechanism but as a central biological program through which cancer cells sustain their expansion and gain metastatic competence [79,80]. This complex process proceeds through a series of highly regulated steps: migration, proliferation, and differentiation of ECs, extracellular matrix (ECM) degradation, and stabilization of new vessels [81].

During the earliest stages of tumorigenesis, passive diffusion is sufficient to meet the oxygen and nutrient exigencies of the small neoplastic mass. However, as the tumor exceeds a critical volume, around 1–2 mm^3^, this mechanism becomes inadequate. The resulting microenvironment becomes progressively hypoxic and nutrient-deprived, with the accumulation of acidic metabolic byproducts. In response, tumor cells stabilize and activate hypoxia-inducible factors (HIFs), a family of transcriptional regulators that orchestrate cellular adaptation to reduced oxygen availability [80]. Once stabilized, HIFs translocate to the nucleus, where they induce the expression of a broad set of pro-angiogenic genes—most notably vascular endothelial growth factor (VEGF)—which acts on ECs, initiating what is considered the predominant mechanism underlying tumor angiogenesis: vessel sprouting [82]. Specifically, VEGF binds to its principal receptor, VEGFR-2, on the surface of ECs, triggering intracellular signaling pathways such as PI3K/AKT and p38/MAPK, which promote their proliferation, migration, and increased vascular permeability. In parallel, ECs acquire two distinct phenotypes: tip cells, which migrate toward hypoxic zones following VEGF gradients, and stalk cells, which proliferate to elongate the developing vascular sprout [83]. This process occurs concurrently with ECM remodeling, facilitating sprout extension.

Subsequently, anastomosis occurs, whereby sprouts from adjacent vessels connect, leading to the formation of new vascular lumens. This culminates in the establishment of a complex and functional vascular network composed of capillaries, arterioles, and venules, thereby sustaining both the expansion and metabolic requirements of the growing tumor mass [83,84].

Once the initial vascular scaffold has been established, ECs initiate the transition from sprouting to stabilization by releasing a repertoire of signaling molecules that initiate the recruitment and functional engagement of pericytes. Among these, transforming growth factor-β1/2 (TGF-β1/2) promote the chemotactic recruitment of pericytes to the developing sprout, while heparin-binding EGF-like growth factor (HB-EGF) enhances their proliferation, collectively contributing to the mechanical reinforcement of the nascent vasculature [85]. In parallel, activated ECs secrete platelet-derived growth factors (PDGFs), which not only recruit pericytes, but also engage cancer-associated fibroblasts (CAFs) from the surrounding stroma, further supporting the structural maturation of the vessel wall. PDGFs act through PI3K and RAS/MAPK signaling pathways to promote vessel stabilization, while specific isoforms such as PDGF-AB and PDGF-BB modulate VEGFR-2 activity and endothelial proliferation, thereby preventing disorganized vascular growth often induced by excessive VEGF signaling [86–89].

Importantly, pericytes are not merely passive responders to endothelial cues but function as active regulators of vessel homeostasis, exerting a pivotal role in guiding the angiogenic process toward maturation and stabilization. They are a principal source of angiopoietin-1 (Ang-1), a key ligand that binds to the Tie2 receptor on ECs and triggers downstream signaling cascades involving Calpain, AKT, and FOXO3A. This pathway suppresses endothelial proliferation, reinforces intercellular junctions, and promotes vessel quiescence. By antagonizing the destabilizing effects of VEGF and Ang-2, Ang-1/Tie2 signaling facilitates the transition from a proliferative to a stabilized vascular phenotype [90].

Although angiogenesis has long been recognized as a hallmark of solid tumor biology, accumulating evidence suggests that it also plays a significant role in the pathogenesis and progression of lymphomas [91]. Its involvement in primary CLs is gaining increasing attention, although current data remain limited and preliminary, warranting further investigation.

## Angiogenesis and lymphangiogenesis in CTCLs

Angiogenesis appears to play a pivotal role in the pathogenesis and progression of CTCLs, impacting not only vascular remodeling, but also the biological behavior of malignant T cells. Early studies have shown that several pro-angiogenic mechanisms—including the VEGF/VEGFR axis, the CXCR4/CXCL12 chemokine pair, and the JAK3/STAT5 signaling pathway—promote tumor cell proliferation, homing, survival, and dissemination through autocrine feedback loops. As the disease advances, malignant T cells themselves begin to produce a variety of angiogenic and lymphangiogenic mediators, such as podoplanin, lymphatic vessel hyaluronan receptor-1 (LYVE-1), VEGF-A, VEGF-C, and lymphotoxin alpha (LTα), which in turn stimulate IL-6 expression and promote both angiogenesis and lymphangiogenesis [28,81,92–94]. These findings support the existence of a self-amplifying circuit that sustains the pro-tumoral microenvironment, as LTα, IL-6, and VEGF appear to drive endothelial expansion and tumor spread. The tumor stroma further enhances this loop by releasing additional angiogenic signals, which not only support neovascularization, but may also reinforce the expression and activity of VEGF within the microenvironment [95]. Among these mediators, VEGF-A primarily binds VEGFR-1 and VEGFR-2 on ECs to induce vascular proliferation and permeability, while VEGF-B signals through VEGFR-1, and VEGF-C and VEGF-D promote lymphangiogenesis via VEGFR-3, with additional angiogenic effects through VEGFR-2. These ligands form a complex network of molecular interactions that link malignant T cells and endothelial compartments. Indeed, VEGF expression in neoplastic T cells has been associated with constitutive activation of JAK3 and c-Jun N-terminal kinases (JNKs), detectable even in early stages of CTCLs, indicating a key role in vascular remodeling and disease progression [96]. Moreover, VEGF-C levels result to be elevated in advanced-stage MF and correlate with increased podoplanin expression, suggesting their potential value as markers of more aggressive clinical phenotypes [75,76,81]. Immunohistochemical evidence further indicates that VEGF-A overexpression in MF is largely driven by the malignant T cells themselves, unlike in reactive or inflammatory conditions, pointing to its role as an intrinsic feature of tumor initiation [97].

Furthermore, a correlation between angiogenic activity and disease stage in CTCLs has been observed. Microvessel density (MVD), assessed via von Willebrand factor expression, and microvessel count, evaluated through CD34 or CD31 immunostaining, are significantly elevated in lesional skin from patients with plaque- or tumor-stage MF, as well as in advanced MF (stage III and IV). It is reasonable to infer that this vascular expansion reflects both increased tumor burden and disease progression [98,99]. Elevated serum and lesional levels of angiogenin, a potent angiogenic mediator, have been associated with erythrodermic CTCLs, including SS. Specifically, angiogenin is expressed by both neoplastic T lymphocytes infiltrating the dermis and/or by tumor-associated host cells present within the lesional skin [99,100]. Similarly, serum levels of Ang-2, a dual regulator of angiogenesis and lymphangiogenesis, are elevated in patients with SS and correlate with disease severity [101]. Likewise, an increase in lymphangiogenesis is observed, as evidenced by upregulated podoplanin expression, which is associated with shorter overall survival in CTCL patients. Moreover, high expression of both endothelial and lymphatic markers has been linked to lymph node involvement [102,103].

Additionally, placental growth factor (PlGF) has also been investigated, adding further complexity to the overall angiogenic landscape in CTCLs. Indeed, although PlGF is known to act synergistically with VEGF-A in promoting cutaneous angiogenesis [104,105], its specific role in patients with MF/SS remains poorly defined. To clarify this, Miyagaki et al. [106] studied PlGF expression by measuring its levels in both lesional skin and serum of MF/SS patients, comparing them to VEGF-A expression. The study revealed a significant upregulation of both VEGF-A and PlGF mRNA and protein in lesional skin compared to healthy controls. Furthermore, these elevated levels positively correlated with disease severity, particularly in advanced stages (IIB and IVA1), aligning with previous reports that PlGF and VEGF-A may cooperatively enhance angiogenesis [104,105]. In addition, serum PlGF concentrations were elevated in patients with advanced MF/SS and showed a positive correlation with key disease biomarkers, including not only Ang-2, but also CCL27, a chemokine involved in T cell skin homing and interleukin-10 (IL-10), an immunosuppressive cytokine known to contribute to tumor immune evasion. The coordinated upregulation of these molecules alongside PlGF suggests the existence of an angiogenic and immunoregulatory network that may facilitate tumor progression in CTCLs [106].

In addition to classical angiogenic pathways, emerging evidence from in vitro studies suggests that alternative pro-angiogenic mediators may also contribute to the angiogenic landscape of CTCLs and their complex microenvironment. Among these, interleukin-17F (IL-17F) has drawn attention for its ability to enhance endothelial sprouting and tube formation—two critical steps in the angiogenic cascade. Its expression appears to be driven by aberrant activation of the JAK3/STAT5 signaling pathway in malignant T cells, resulting in its constitutive secretion [52,107]. Furthermore, elevated IL-17F gene expression has been associated with poor prognosis, possibly due to its ability to modulate stromal cell function and promote tumor progression [96,107–109]. Based on these observations, Lauenborg et al. proposed a potential role for IL-17F in MF, particularly in patients exhibiting elevated levels of IL-17A and/or IL-17F. They demonstrated that IL-17F derived from malignant T cell lines promotes endothelial morphogenesis. Notably, these same malignant T cells also express LTα, whose production is induced by dysregulated JAK3/STAT5 signaling, thereby contributing to IL-6 secretion and synergizing with VEGF to support tumor vascularization [52,93]. In parallel, neoplastic T cells in early-stage MF have been reported to exhibit increased CXCR4 expression, which, through interaction with its ligand CXCL12, establishes a signaling axis that facilitates both malignant cell trafficking and angiogenesis, ultimately promoting disease progression [110].

**Fig. 1:**
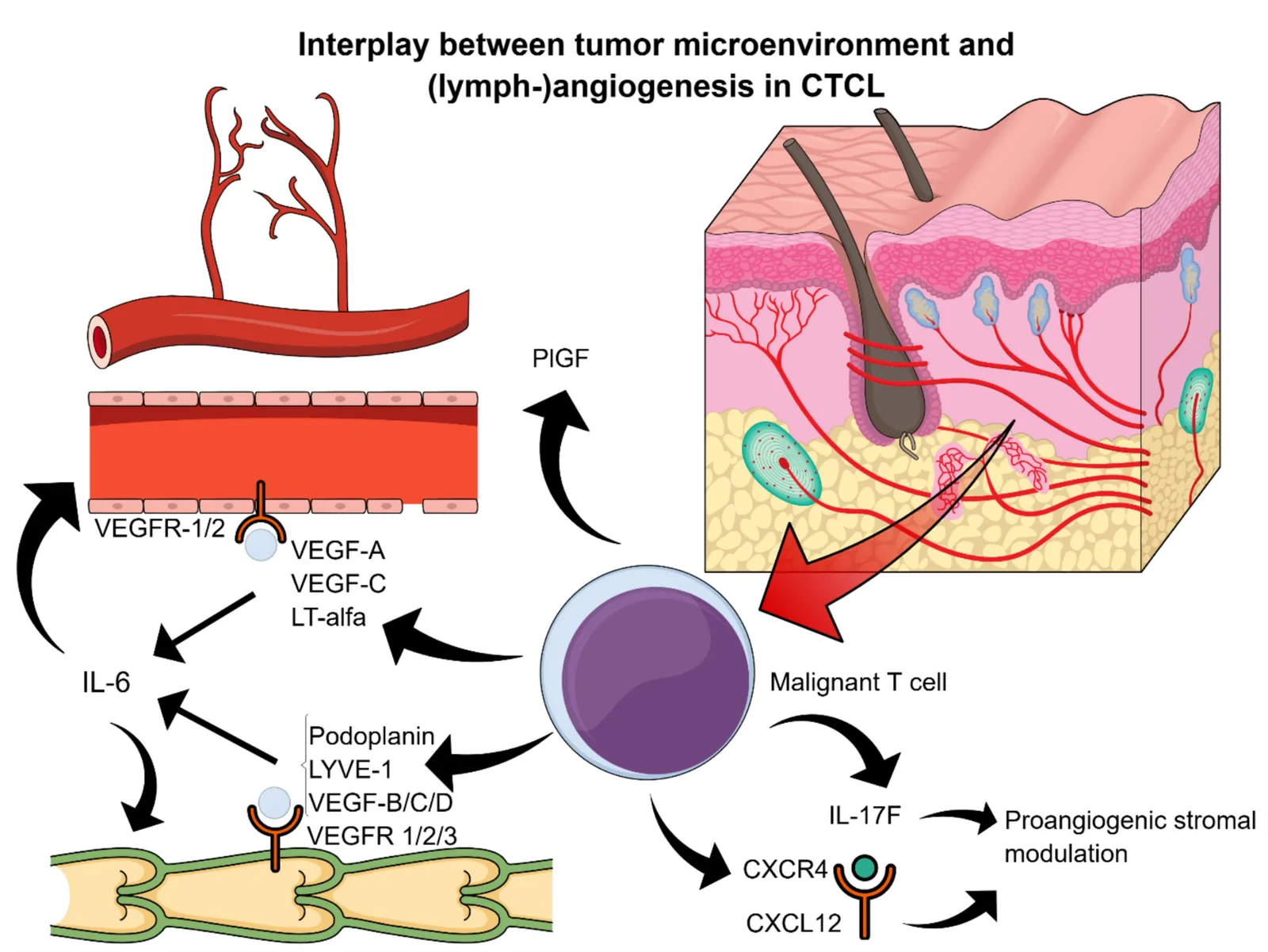
Summary of angiogenic and lymphangiogenic pathways in CTCLs

Taken together, these findings underscore the multifaceted contribution of angiogenesis and lymphangiogenesis to CTCLs pathobiology, impacting not only vascular architecture, but also tumor growth, immune modulation, and clinical behavior. However, much of the available evidence—though compelling—remains preliminary, with many insights derived from in vitro studies or limited patient samples. Additional pro-angiogenic mediators such as IL-17F, PlGF, and the CXCL12/CXCR4 axis have shown potential relevance, but require further investigation to define their mechanistic roles and validate their significance in larger clinical cohorts. It is therefore evident that more studies are warranted to clarify the causal relationships, spatial and temporal dynamics, and biological consequences of these angiogenic pathways in CTCLs. Crucially, a more comprehensive understanding of the angiogenic landscape in this setting could pave the way for the development of novel therapeutic strategies. Whether modulation of tumor-associated vasculature will yield meaningful clinical benefit in CTCLs—as it has in solid malignancies—remains an open question of considerable translational relevance. Figure 1 summarizes the most important angiogenic pathways involved in the pathobiology of CTCLs.

## Angiogenesis in CBCL

In contrast to the more studied role of angiogenesis in CTCLs, its contribution to the biology and progression of CBCLs is less clearly defined. Early observations by Schaerer et al. [111] revealed a significant higher MVD in CBCLs compared to pseudolymphomas, suggesting a true neoplastic angiogenic switch. Expanding on this, Wobser et al. [112] employed immunohistochemical analysis to determine whether prognostically different subtypes of CBCLs could be distinguished based on the evaluation of MVD, as well as the expression of VEGF. The results showed that intratumoral MVD was comparable among the indolent subtypes (PCFCL, PCMZL), whereas the highest density was observed in the more aggressive PCLBCL-LT. Notably, this increased MVD correlated with higher tumor cell positivity for VEGF, particularly in lesions localized to the lower extremities. Of note, in this study, VEFG expression was not only restricted to lymphoma cells, but also various stromal cells within the TME stained positive, underlying the importance of the microenvironment for the angiogenic response [113]. Moreover, given that VEGF may exert an autocrine effect within the TME, the expression levels of VEGFR-1 and VEGFR-2 were also assessed. Both receptors were found to be more highly expressed in PCLBCL-LT, suggesting a potential role in promoting tumor angiogenesis and progression in this specific subtype. Teichert et al. [114] also studied PCLBCL-LT, highlighting the role of Ang-2 as a key driver of angiogenesis in PCLBCL-LT, showing that its expression is significantly upregulated compared to indolent CBCLs. A critical finding of this study is the dysregulation of the Ang-1/Ang-2 balance, where Ang-2 is markedly elevated in the peripheral blood of PCLBCL-LT patients. Unlike Ang-1, which promotes vascular stability through its interaction with the Tie2 receptor, Ang-2 leads to endothelium activation and vascular remodeling. Interestingly, ECs within PCLBCL-LT tumors exhibit reduced expression of the Tie2 receptor, a phenomenon that shifts Ang-2 signaling toward an integrin-mediated angiogenic pathway. This alternative signaling mechanism enhances focal adhesion kinase (FAK) phosphorylation, driving vessel sprouting and increased MVD within the tumor microenvironment. These findings suggest that angiogenesis contributes to the disease biology of aggressive CBCLs, particularly PCLBCL-LT, where both VEGF/VEGFR and Ang-2/integrin-FAK pathways appear to drive pathological neovascularization. Ang-2 may promote vascular growth independently of Tie2, highlighting an alternative pro-angiogenic route. The association between high MVD, VEGF/Ang-2 expression, and tumor aggressiveness points to angiogenesis as a potential biomarker and therapeutic target. However, these insights remain preliminary and require further validation before clinical translation.

## Therapeutic approaches

The study of the possible inhibition of angiogenesis (e.g., anti-angiogenesis) has always attracted a certain interest in the scientific community, and in the field of CTCLs, attempts have been made to conduct research. Being central to the angiogenic pathway, the VEGF/VEGFR axis is of some importance, with potential research interest regarding the use of Bevacizumab (anti-VEGF), as well as the use of VEGF-Trap and VEGF-antisense [95]. In detail, to date, the information we have regarding the use of Bevacizumab concerns pre-clinical, experimental models without clinical studies regarding its use in PCLs. A work has hypothesized that the depletion of mast cells in the TME of PCLs could increase the anti-angiogenic efficacy of anti-VEGF drugs, but these data will need to be confirmed with controlled and randomized clinical studies [22,115]]. Regarding Aflibercept (VEGF-Trap), to date, there are no clinical studies involving patients affected by PCLs, although recognizing the importance of the molecular pathway inhibited by this molecule. Some clinical trials have been conducted on patients with systemic B cell lymphomas in combination with Rituximab-Cyclophosphamide-Hydroxydaunorubicin-Oncovin-Prednisone (R-CHOP) [115]. Regarding VEGF-antisense (ASO), a phase I trial was conducted in 2003/2004 that included patients with previously treated refractory tumors, including some with CTCL. Veglin (the name of the ASO) was administered to 35 patients, including a subset with CTCL, and a reduction in plasma levels of VEGF-A was observed in 47% and VEGF-C in 21%, with cases with regression of skin lesions for several months and a partial response in at least one CTCL patient [116]. Regarding the use of immunomodulatory drugs with anti-angiogenic properties, Thalidomide has been used in limited cases and small trials, in patients with PCLs. In a 2006 paper, an isolated case showed response to Thalidomide in chemotherapy-resistant CTCL [117], but there are no recent specific trials on MF or CTCL with good controlled evidence. Lenalidomide (Revlimid) exerts a more potent anti-angiogenic effect [118]. An EORTC 21081 phase III study compared maintenance with lenalidomide vs observation after “debulking” therapy in 30 registered and 21 randomized patients. Median progression-free survival was 5.3 months vs 2 months, with non-significant improvement for a limited number of patients [119]. Pomalidomide (Pomalyst/Imnovid) also has a more potent anti-angiogenic/immunomodulatory effect in vitro [121] but no clinical trials specifically targeting cutaneous lymphomas have been identified. The antiregulatory activity (on T-reg) supports the biological rationale, but clinical data applied to CTCL are lacking. Finally, Apremilast (Otezla), an oral PDE4 inhibitor, reduces pro-inflammatory cytokines and modulates T cells/NK and is typically used in psoriasis and psoriatic arthritis, but there is no current clinical evidence for its use in cutaneous lymphomas. Some pre-clinical studies suggest a potential immunomodulatory mechanism of action, but further research is needed.

In addition to drugs that inhibit the JAK/STAT pathway (JAKi), attention has also been paid to epigenetic drugs, among which Resminostat, an inhibitor of histone deacetylases (HDACi), stands out. A first attempt to understand the anti-angiogenic efficacy of the ruxolitinib–resminostat combination was performed on chick embryo (CAM) models (models for the study of angiogenesis) and further developed recently [56,111]. Indeed, this last part of research investigates the synergistic therapeutic effects of ruxolitinib and resminostat, through in vivo and in vitro models, using two recognized human CTCL cell lines, MyLa and SeAx, representing distinct stages of the disease. (MyLa line was derived from plaque-stage MF, while SeAx originates from Sézary Syndrome (SS).) The researchers investigated the impact of the combination on cell migration, angiogenesis, gene expression, and signaling pathways and, to make this one, cells were administered with ruxolitinib (15 μM) and resminostat (10 μM), followed by several assays to evaluate the effects of monotherapy compared to combination therapy. A transendothelial migration experiment utilizing human vascular endothelial cells (HVECs) shows that only the combination therapy markedly decreased CTCL cell migration—by 75% in MyLa cells and 82% in SeAx cells—while monotherapies exhibited no effects, indicating a synergistic impact that inhibits CTCL cell migration across endothelial barriers, which is essential for tumor propagation. The CAM experiment revealed that the combination treatment markedly decreased angiogenic blood vessel formation—by 49% in MyLa and 34% in SeAx—relative to the control group; furthermore, the combination inhibited pro-angiogenic VEGF-A gene expression by 61% in MyLa and 79% in SeAx, while simultaneously upregulating anti-angiogenic VEGF-B expression by 49% and 56%, respectively. Hemoglobin quantification indicated less vascularization in treated embryos, especially in SeAx cells. Finally, RT-PCR analyses of gene expression demonstrated that combination therapy did not influence HDAC gene expression; nevertheless, HDAC enzymatic activity was significantly inhibited, especially with resminostat or the combination, so affirming epigenetic regulation. Western blot investigations demonstrated that the combination markedly downregulated critical signaling molecules, such as phospho-AKT, phospho-ERK, and phospho-STAT5, which are essential for tumor survival, proliferation, and angiogenesis.

The combination markedly diminished tumor mass in both CTCL models, with reductions of 49% in MyLa and 61% in SeAx, and resminostat monotherapy had a modest yet significant anti-angiogenic effect in the SeAx model, presumably linked to its correlation with more aggressive illness.

The study finds also that monotherapies are inadequate for effectively modifying angiogenesis and tumor progression in CTCL, but the combination of ruxolitinib and resminostat exhibits significant synergistic effects on critical tumor-promoting pathways. This dual-targeting method may effectively manage CTCL by decreasing angiogenesis, migration, and tumor growth through the downregulation of VEGF-A, reduction in HDAC activity, and inhibition of JAK/STAT, PI3K/AKT, and ERK signaling pathways [56].

## Concluding remarks

Neoangiogenesis and lymphangiogenesis play potentially important pathogenic roles in CL initiation and prognostic, by stimulating homing of tumor cells and generation of pro-proliferative soluble cues. The main signaling pathway involved in autocrine stimulation of proliferation and survival of lymphoma tumor cells is VEGF-VEGF receptor axis, although other axes, described in homing studies (CXCR4/CXCL12) or signaling pathways (JAK3/STAT5), are also involved. The anti-angiogenic therapy is an important tool for the treatment of CLs. However, a significant number of patients are resistant, whereas those who respond have minimal benefits. A tumor resistance and significant side effects including toxicity can occur. Further research should provide new useful therapeutic approaches and increase options for patients with resistant or refractory disease. Further understanding of the CL-specific and drug-specific mechanisms underlying anti-angiogenic therapy is fundamental to reach a better understanding of the complex biology of lymphoma angiogenesis and validation of clinically useful biomarkers which reflect the dynamics of drug and target interaction.

## Data Availability

No datasets were generated or analysed during the current study.
